# [Retracted] Ruscogenin induces ferroptosis in pancreatic cancer cells

**DOI:** 10.3892/or.2025.8863

**Published:** 2025-01-03

**Authors:** Zhiwang Song, Xiaojun Xiang, Junhe Li, Jun Deng, Ziling Fang, Ling Zhang, Jianping Xiong

Oncol Rep 43: 516–524, 2020; DOI: 10.3892/or.2019.7425

Following the publication of this paper, it was drawn to the Editor's attention by a concerned reader that certain of the western blot data shown in Fig. 4 on p. 521 were strikingly similar to data that had already appeared in a pair of figures in a previously published article written by different authors at different research institutes in the journal *Molecular Cancer Therapeutics*. In addition, there was an unexpected issue with the appearance of the shading surrounding one of the tumor images featured in Fig. 7, such that this figure appeared to contain an anomaly.

Owing to the fact that contentious western blot data in the above article had already been published prior to its submission to *Oncology Reports*, the Editor has decided that this paper should be retracted from the Journal. The authors were asked for an explanation to account for these concerns, but the Editorial Office did not receive a reply. The Editor apologizes to the readership for any inconvenience caused.

